# Comparison of Water Sorption and Water Solubility Properties of Current Restorative Materials with Different Contents

**DOI:** 10.1055/s-0044-1789270

**Published:** 2024-09-18

**Authors:** Magrur Kazak, Tugba Toz Akalin, Fevzi Esen

**Affiliations:** 1 Department of Restorative Dentistry, Bahcesehir University School of Dental Medicine, Istanbul, Türkiye; 2 Department of Therapeutic Dentistry, BAU International University School of Medicine and Health Science, Batumi, Georgia; 3 Department of Restorative Dentistry, Istinye University Faculty of Dentistry, Istanbul, Türkiye; 4 Department of Health Information Systems, University of Health Sciences, Institution of Hamidiye Medical Sciences, Istanbul, Türkiye

**Keywords:** alkasites, bulk-fill composite resin, glass hybrid, water solubility, water sorption

## Abstract

**Objectives** This study aimed to investigate and compare water sorption and solubility properties of current restorative materials with different contents.

**Materials and Methods** Alkasite, self-adhesive restorative material (Cention N, Ivoclar Vivadent AG, Schaan, Liechtenstein), bulk-fill glass hybrid restorative material (EQUIA Forte HT, GC Corp., Tokyo, Japan), nanohybrid universal composite material (OptiShade, Kerr Dental, United States), and bulk-fill composite material (Filtek One Bulk Fill Restorative, 3M ESPE, St. Paul, Minnesota, United States) were used. Samples ( *n =* 6) were prepared (2 × 10 mm) according to the ISO 4049 standards. Water sorption and solubility values were calculated according to the ISO 4049 standards.

**Statistical Analysis** One-way ANOVA, Tukey's post-hoc, Tamhane's T2 post-hoc, Pearson's correlation, and independent samples *t* -tests were used for statistical analysis ( *p <* 0.05).

**Results** Group EQUIA Forte HT significantly showed the highest water sorption values (57.278 ± 3.174), while Group Filtek One Bulk Fill Restorative exhibited the lowest (4.429 ± 0.174; *p <* 0.05). The water sorption values for Group Cention N were 5.000 ± 0.542. Group EQUIA Forte HT significantly had the lowest water solubility values (−99.799 ± 1.909), while Group Cention N (−2.966 ± 0.402) significantly exhibited the highest ( *p <* 0.05). There was no significant correlation between water sorption and solubility values for each material ( *p >* 0.05).

**Conclusion** The bulk-fill nano-filled composite resin material was successful in terms of water sorption while the bulk-fill glass hybrid restorative system in terms of water solubility. Alkasite can be recommended to be used as a base material due to its high solubility feature. Monomer, filler type, and amount had an impact on the water sorption and solubility properties of the tested materials.

## Introduction

To phase out amalgams as a result of the Minamata Agreement, composite resin materials have been developed as an alternative to dental amalgam in the posterior region, with many superior properties. 1 However, the application of conventional composite resin materials in incremental layers to reduce the stress and amount of polymerization shrinkage increases the polymerization steps, especially in deep and large cavities, which is time-consuming for both patients and clinicians. To overcome this problem, bulk-fill composite resin materials have been developed to enable the composite resin materials to be applied in larger masses like 4 to 5 mm. The feature that allows the bulk-fill composite material group to be placed in bulk is that chemical groups with polymerization modulators, which can reduce polymerization shrinkage stress and allow light to reach the deepest parts of the cavity, are located in the resin matrix structure of the material. 2 3 4 5 6 Bulk-fill materials can be classified as low and high viscosity. 2 High-viscosity bulk-fill composites contain more filler and therefore exhibit higher mechanical properties than low-viscosity (flowable) bulk-fill materials. Therefore, high-viscosity bulk-fill composites can be successfully used in large restorations of posterior teeth. 7

In current clinical practice, also glass ionomer-based restorative materials are widely used. High-viscosity glass ionomer cement was developed with enhanced mechanical properties, providing long-lasting restorations for the posterior Class I and II cavities. 8 The material has its latest version since 2019 which is considered a glass-hybrid material, EQUIA Forte HT. The optimized particle size and distribution have improved the material's strength, translucency, and aesthetic properties. 9

Recently, another type of restorative material was introduced for bulk placement in retentive cavities. This material is a self-adhesive, dual-cured resin-based bulk-fill composite with alkaline fillers, referred to as alkasites (Cention N). 10 It was pointed out that the clinical performance of Cention N was similar to that of a bulk-fill resin composite in Class I and two-surfaced Class II restorations. 11

Besides the meticulous application of proper protocols and clinical techniques, one of the clinical success of restorations is dependent on the selection of the appropriate restorative material. Mechanical properties such as water sorption and solubility give a considerable idea about the long-term clinical performance of a material 12 13 and affect all restorative materials' physical, mechanical, and biological properties. 14 Therefore, clinicians should choose the appropriate material while performing a restoration.

Water sorption is a diffusion-controlled, time-dependent process that can reduce the life of the material by expanding and plasticizing the resin component and hydrolyzing the silane. 15 This causes dimensional changes in the material, resulting in discoloration and fractures at the restoration edges. 16 In the water sorption process, fluids enter the structure of the material by diffusion, initiating the elution of free residual monomers from the resin structure, causing chemical degradation and dissolution in water. 17 18 In addition, the elution of the residual monomers can have a negative impact on biocompatibility. 19 20 In conclusion, restorative materials that do not have ideal water sorption and solubility values can lead to deterioration of marginal integrity and surface properties, resulting in loss of aesthetic appearance. 16 19 20

Studies investigate the water sorption and solubility properties of restorative materials with different contents. 14 21 22 23 However; there is no study in the same article evaluating the water sorption and solubility of alkasite self-adhesive restorative material, bulk-fill glass hybrid restorative material, nanohybrid composite material, or bulk-fill composite resin material.

Accordingly, the first aim of this *in vitro* study was to investigate and compare the water sorption and water solubility properties of current restorative materials with different contents used in the posterior region. The second aim was to evaluate the correlation between each material's water sorption and water solubility values.

- The first null hypothesis of the study is that the current restorative materials will exhibit lower water sorption and solubility values.
- The second null hypothesis is that there will be a correlation between each material's water sorption/water solubility values.

## Materials and Methods

The restorative materials evaluated in the study, including the types, contents, and application procedures, are listed in Table 1 .

**Table 1:** Restorative materials used in the study with types, contents, and application/preparation procedures

Material/manufacturer	Type	Content	Preparation procedure
**Cention N (CN)** (Ivoclar Vivadent AG, Schaan/Liechtenstein) Shade: A2 (LOT Number: Z0233Y)	Alkasite, self-adhesive restorative material	***Powder:*** Barium aluminosilicate glass (20–30%), ytterbium trifluoride (5–10%), isofiller (15–25%) (Tetric N-Ceram technology), calcium barium aluminofluorosilicate glass, calcium fluorosilicate (alkaline) glass (25–35%) Filler content by weight 78.4%/by volume 57.6% Filler size: 0.1–35 µm. ***Liquid:*** UDMA, DCP, aromatic aliphatic-UDMA, PEG-400 DMA *Photo-initiator:* ivocerin, hydroperoxide, and acyl phosphine oxide	A measuring spoon of powder and a drop of liquid are mixed until it reaches a smooth consistency. Mixing time should not exceed 45–60 seconds. Working time is 3 minutes from the start of mixing. Self-setting time is 5 minutes from the start of mixing. The light-curing is 20 seconds.
**EQUIA Forte HT (EF)** (GC Corp., Tokyo Japan) Shade: A2 (LOT Number: 210504A) **EQUIA Forte Coat** (LOT Number: 2104211)	Bulk-fill glass hybrid restorative system Light-cure resin coating agent	***Powder*:** 95% strontium fluoroaluminosilicate glass, 5% polyacrylic acid, iron oxide ***Liquid:*** polybasic carboxylic acid (tartaric acid) 5–10% by weight and water	The capsule is mixed in the amalgamator for 10 seconds. The capsule is then attached to the applicator and applied to the cavity for 10 seconds. The working time of the material is 90 seconds. Finally, Equia Forte coat is applied and light cured for 20 seconds.
**OptiShade (OS)** (KaVo Kerr, United States) Shade: MD (Medium) (LOT Number: 8242079)	Nanohybrid universal composite restorative material	***Organic Matrix:*** Bis-GMA, Bis-EMA, TEGDMA *** Inorganic Matrix:*** mixed oxides, prepolymerized filler, barium aluminosilicate glass, silica and ytterbium trifluoride Adaptive response technology (ART) Filler content by weight 81.5%/by volume 65.1% Smallest primary particle size: 5 nm, largest primary particle size: 400 nm, average particle size: 50 nm	The light-curing is 20 seconds.
**Filtek One Bulk Fill Restorative (FO)** (3M ESPE, St. Paul, Minnesota, United States) Shade: A2 (LOT Number: NC17470)	Bulk-fill nano-filled composite resin material	***Organic Matrix:*** AUDMA, UDMA, AFM, DDDMA ***Inorganic Matrix:*** 20 nm silica, 4–11 nm zirconia, zirconia/silica clusters, 100 nm ytterbium trifluoride fillers Filler content by weight 76.5%/by volume 58.4%	The light-curing is 20 seconds.

Abbreviations: AFM, addition fragmentation monomer; AUDMA, aromatic urethane dimethacrylate; Bis-EMA, ethoxylated bisphenol; Bis-GMA, bisphenol A-glycidyl methacrylate; DCP, tricyclodecane dimethanol dimethacrylate; Aromatic Aliphatic-UDMA, tetramethyl-xylylene diurethane dimethacrylate; DDDMA, 1,12-dodecane dimethacrylate; PEG-400 DMA, polyethylene glycol 400 dimethacrylate; TEGDMA, triethylene glycol dimethacrylate; UDMA, urethane dimethacrylate.

## Specimen Preparation

An alkasite, a self-adhesive restorative material (Cention N, Ivoclar Vivadent AG, Schaan, Liechtenstein) (CN), a bulk-fill glass hybrid restorative material (EQUIA Forte HT, GC Corp., Tokyo, Japan) (EF), a nanohybrid universal composite material (OptiShade, Kerr Dental, United States) (OS), and a bulk-fill composite material (Filtek One Bulk Fill Restorative, 3M ESPE, St. Paul, Minnesota, United States) (FO) were used in the study. Six samples ( *n =* 6) from each material were prepared according to the ISO 4049 standards 24 and then placed in cylindrical Teflon molds with a height of 2 mm and a diameter of 10 mm. 22 These molds were placed between two Mylar strips with two glasses to obtain a flat surface. Groups Cention N, OptiShade, ^,^ and Filtek One Bulk Fill Restorative were polymerized using an LED light-curing device (Demi Ultra, LED Ultracapacitor, Kerr, United States) positioned on the top surface with a light intensity of 1,100 mW/cm ^2^ . The samples received a radiant exposure of 22 J/cm ^2^ . 25 26 According to ISO 4049 (ISO-Standards 2009) 24 only the sides of the samples belonging to these three groups were smoothed with polishing discs (Sof-Lex, 3M ESPE, St. Paul, Minnesota, United States). The top surfaces of the Group EQUIA Forte HT were only polished with polishing discs (Sof-Lex, 3M ESPE, St. Paul, Minnesota, United States) from coarse (10,000 RPM) to fine (30,000 RPM) five times in the same direction. 24 Care was taken not to apply pressure to the samples and polishing was simulated as if working on a patient's tooth. At each disc change, the samples were rinsed with water and dried. A polishing disc was replaced with a new one in every two samples. Then EQUIA Forte Coat was applied over the samples and light-cured for 20 seconds by an LED light-curing device (Demi Ultra, LED Ultracapacitor, Kerr, United States) with a light intensity of 1,100 mW/cm ^2^ .

Before the polymerization of each experimental group, the battery level and light intensity of the light source were checked with a radiometer (LED Radiometer, SDI Ltd., Australia) to provide standardization. Attention was given to touching the tip of the light-curing device to the surface of the glass.

## Water Sorption and Water Solubility Measurements

The samples were kept in dark-colored glass bottles on which the caps were not completely closed, placed in a desiccator (Vacucell, MMM, Germany) and then kept inside a vacuum oven for 22 hours at 37°C ± 1°C. After that, the bottles were removed from the oven and left on a bench for 2 hours at 23°C ± 1°C to complete a 24-hour cycle. Later, the samples were weighed daily with an analytical balance (Precisa, ES 225SM-DR, Switzerland) to record the 24-hour weighing cycles. The complete cycle was repeated every day at the same time until a constant (the loss for each sample was not more than 0.1 mg per 24-hour cycle) mass ( *M* 1) was obtained. To calculate the sample's volume ( *V* ) in mm ^3^ , the diameter and thickness of each sample were measured three times with a caliper. The samples were then placed back in the dark-colored glass bottles and distilled water (20 mL) was added to the samples with manual pipettes. The glass bottles were sealed, placed in the oven (Stuart Orbital Incubator SI500, Bibby Scientific Ltd., United Kingdom) and kept at 37°C ± 1°C for 7 days. After this procedure, all the glass bottles were removed from the oven and kept at 23°C ± 1°C for 2 hours. The samples were removed from the bottles, dried with absorbent paper (15 seconds), and left in a sterile bucket (1 minute). The samples were weighed again to obtain *M* 2. The samples were reconditioned in the desiccator until they reached a constant mass ( *M* 3) with the cycle used for *M* 1. 21 22

The WSP (water sorption) and WSL (water solubility) values of the samples were calculated (µg/mm ^3^ ) according to the formula specified in ISO 4049 (ISO Standards 2009). 24

WSP = ( *M* 2 − *M* 3)/ *V*

WSL = ( *M* 1 − *M* 3)/ *V*

### Statistical Analysis

In calculating the sample sizes, the probability of type 1 error (α = 0.05) and the power of the test (1− β) were considered to be 0.95. Using the GPower 3.1.9.2 program, it was calculated that the total sample size should be at least six. Therefore, the sample size used was six.

The data were analyzed using SPSS, version 24 (SPSS Inc., Chicago, Illinois, United States). The Shapiro–Wilk test was employed for assessing normality assumption. The normality assumption was fulfilled for all cases ( *p >* 0.05). The means of water sorption and water solubility between the materials were compared using one-way ANOVA (analysis of variance). Levene's test was performed for homogeneity of variance. The variances between the materials were equal in water sorption. Tukey's post-hoc test was used for multiple comparisons between the materials. Besides that, the variances were unequal between the materials in water solubility. Welch's test was used to test whether all materials have the same variances. The mean of water solubility was significantly different between the materials, *F*_Welch_ (3,10.198) = 4,379.143, *p* = 0.000. Due to the violation of the homogeneity assumption, Tamhane's T2 post-hoc test was performed to compare group differences. Pearson's correlation test was also used to determine possible correlations between water sorption and solubility. Independent samples *t* -test was used to compare the materials in terms of water sorption and water solubility. All tests were performed with a significance level of 95%.

## Results

The means, standard deviations, and the results of correlation analysis of water sorption and solubility values for each material are shown in Table 2 .

**Table 2:** Means ± SD of water sorption and solubility (μg/mm ^3^ ) for the tested materials

Materials	Water sorption a	Water solubility b	*r*	*p* c
**Cention N (CN)**	5.000 ± 0.542 ^BC^	−2.966 ± 0.402 ^A^	−0.5952	0.213
**OptiShade (OS)**	7.097 ± 0.422 ^B^	−9.716 ± 0.687 ^B^	−0.2579	0.622
**EQUIA Forte HT (EF)**	57.278 ± 3.174 ^A^	−99.799 ± 1.909 ^C^	−0.4063	0.424
**Filtek One Bulk Fill Restorative (FO)**	4.429 ± 0.174 ^C^	−8.324 ± 0.280 ^D^	−0.2140	0.684

a Tukey's post-hoc test.

b Tamhane's T2 post-hoc test.

c Significance of Pearson's correlation coefficient between water sorption and water solubility for each material. Different uppercase letters indicate significant mean differences between materials at the 0.05 level.

**Fig. 1:**
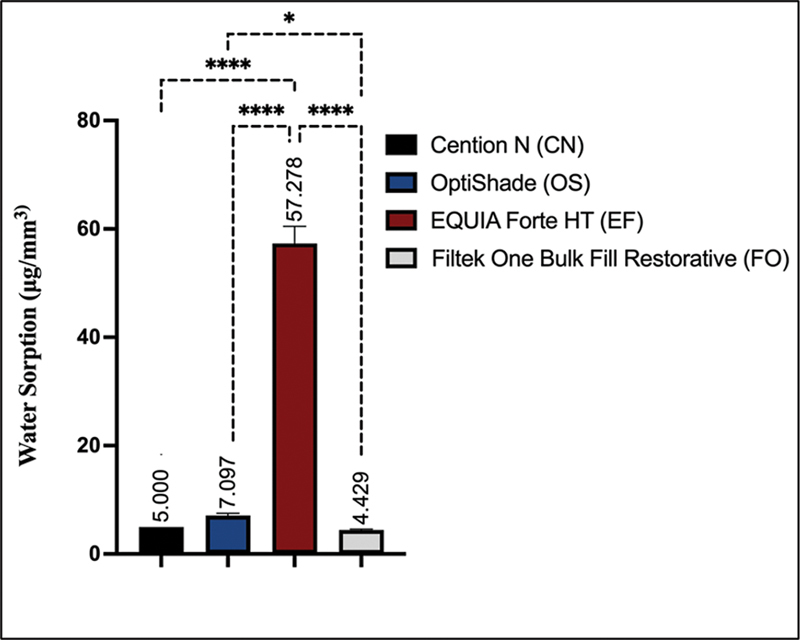
Pairwise comparisons of water sorption values for the tested materials. Note: Nonsignificant pairwise comparisons were not shown in the figure. * *p* < 0.05. **** *p* < 0.0001.

In terms of water sorption values, Group EF significantly showed the highest values (57.278 ± 3.174) compared with the other materials ( *p <* 0.05), followed by OS, CN, and FO, respectively. Group FO had the lowest water sorption (4.429 ± 0.174). A significant difference was observed between groups OS and FO ( *p* = 0.046). However, no significant differences were found between groups CN and FO ( *p* = 0.928), and groups CN and OS ( *p* = 0.148; Fig. 1 ).

**Fig. 2:**
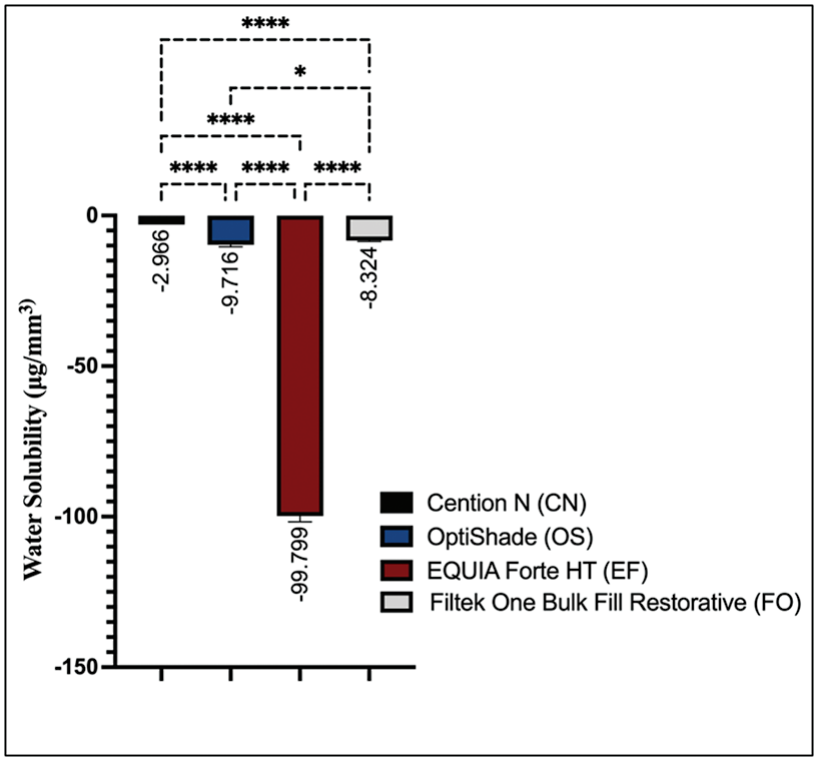
Pairwise comparisons of water solubility values for the tested materials. Note: Nonsignificant pairwise comparisons were not shown in the figure. * *p* < 0.05. **** *p* < 0.0001.

In terms of water solubility values, there were significant differences among all materials ( *p <* 0.05). Group EF significantly had the lowest water solubility values (−99.799 ± 1.909) compared with the other groups ( *p <* 0.05). Group CN (−2.966 ± 0.402) significantly exhibited the highest water solubility values when compared with the other groups ( *p <* 0.05). A statistically significant difference was observed between groups OS and FO ( *p <* 0.05; Fig. 2 ).

According to correlation analysis, there was no significant relationship between water sorption and solubility values for each material ( *p >* 0.05; Table 2 ).

## Discussion

In this study, water sorption and water solubility properties of current restorative materials with different contents used in the posterior region were compared, and the correlation between these two properties was evaluated. According to the ISO 4049 standards, the maximum water sorption value is determined as 40 μg/mm ^3^ , while the maximum water solubility value is 7.5 μg/mm ^3^ . 24 As a result of this *in vitro* study, only EQUIA Forte HT exceeded the maximum sorption value (57.278 ± 3.174). In terms of water solubility, all tested materials' values were lower than the threshold. Therefore, the first null hypothesis is partially accepted. The correlation analysis showed no significant relationship between each material's water sorption/water solubility values ( *p >* 0.05). On that account, the second null hypothesis is rejected.

This *in vitro* study evaluated water sorption and solubility for 1 week (7 days). Although this appears to be a very short time for the materials to reach equilibrium, the 1-week storage time is defined in ISO 4049 standards, 24 and the study is designed to cover this time.

Before starting any restorative procedure, material selection should be considered on a case-based basis in terms of the longevity and clinical success of the restoration. The material should be subjected to various mechanical tests to give an important idea about the long-term clinical performance. Therefore, in this *in vitro* study, current restorative materials used in the posterior region were selected and evaluated concerning water sorption and water solubility.

Since all restorative materials are exposed directly to saliva in the oral environment, water sorption cannot be kept under control completely. 27 Yilmaz et al 28 and Jafarpour et al 29 evaluated the effect of the EQUIA Forte Coat on the water sorption of EQUIA Forte and EQUIA Forte Fil and found the values as 76.70 ± 16.82 and 79.10 ± 15.70, respectively, at the end of 7 days. Savas et al 30 also investigated the water sorption of glass ionomer–based restorative materials and found the value of EQUIA applied with a surface coating as 68.11 ± 13.06 after 7 days. They also emphasized that the maximum amount of water gain occurred during the first week for the hydrophilic materials. In the present study, the water sorption value for Group EF with EQUIA Forte Coat was above the ISO 4049 standards (57.278 ± 3.174) at the end of 7 days and in line with the numerical results of the previous studies. The higher water sorption values obtained from the Group EQUIA Forte HT could be due to the composition of the material, which contains a strontium-based glass and is designed to provide a sustained high release of fluoride ions. This would increase the volume of voids in the material, allowing greater sorption of water. 9

In the present study, the water sorption values of the alkasite self-adhesive restorative material (5.000 ± 0.542), nanohybrid universal composite restorative material (7.097 ± 0.422), and bulk-fill nano-filled composite resin material (4.429 ± 0.174) were below the ISO 4049 standards. It was stated that there is a negative correlation between the amount of filler loading (by weight) and water sorption values. 12 As the filler loading of the material increases, the polymeric matrix phase and water sorption decrease. According to this statement, Group OS, with the highest filler content (81.5%), should exhibit the lowest water sorption value when compared with Groups CN and FO. But besides filler content, the monomer type is also effective on the water sorption values. The water sorption of composite resins is a diffusion-controlled process, and the water uptake takes place mostly within the resin matrix. 31 In the study of Sideridou et al 32 the water sorption values of the monomers were compared, and they were listed from low to high as Bis-EMA < UDMA < Bis-GMA < TEGDMA. According to Ortengren et al, 18 hydrophilic monomers such as TEGDMA and Bis-GMA were more responsible for the increase in sorption values. In the organic matrix part of Group OS, both TEGDMA and Bis-GMA were included, which could explain the higher water sorption values when compared with the values for groups CN and FO. The filler type is also effective in terms of water sorption. Electropositive metal ions (barium and zinc) including materials tend to have greater reactivity with water and may have more hydrolytic degradation. 33 The presence of barium ions in the groups CN and OS provides hydrophilicity to these materials, making them more susceptible to water sorption. Although Group FO had the least filler content (76.5%), this group showed the least water sorption value (4.429 ± 0.174). This can be attributed to the monomer types (hydrophobic monomer UDMA) and inorganic particles which make the material show better mechanical properties in terms of water sorption.

In this study, the water solubility values of all tested materials were lower than the ISO 4049 standards (7.5 mg/mm ^3^ ) and exhibited negative values. The first explanation for the negative solubility values is that there was incomplete dehydration of the materials. This does not mean that no solubility or eluate was produced from these materials; in contrast, there was a low solubility. 18 The explanation is agreed by the current authors and the findings of the present study are in line with the first explanation. The second explanation for the negative values is these materials were more suitable for water sorption. Fabre et al 34 stated that the water sorption capacity was greater than the solubility; therefore, sorption could have masked the actual solubility. Besides Alshali et al 12 mentioned that dental materials with higher sorption values do not necessarily demonstrate greater solubility and vice versa. Therefore, the current authors defend the statement of Alshali et al 12 and can partially accept the second explanation. The second explanation was valid only for Group EF, which exhibited the least solubility (−99.799 ± 1.909) and the highest sorption value (57.278 ± 3.174).

Cention N is a self-curing filling material with a light-curing option. 35 To standardize the sample preparation protocol for all tested materials, it was preferred to polymerize the samples for Group CN in this study. Although Group CN and Group EF are both called ion-releasing materials, they did not show similar water solubility values. The highest water solubility value was detected for Group CN (−2.966 ± 0.402), while the lowest was for Group EF (−99.799 ± 1.909). The highest water solubility values for Group CN could be related to one of the hydrophilic liquid monomers, PEG-400 DMA, in the content of the material which is capable of releasing ions. 36 During the release of ions from the surface of the material, the loss of mass and evident porosities will happen, and this situation will accelerate the solubility of the material.

Although groups OS and FO exhibited similar water solubility values, a statistically significant difference was obtained between these groups ( *p <* 0.05). The similar solubility values of these materials could be attributed to being the same material type, composite resin, that they fall under.

In the present study, the correlation was investigated and according to the correlation analysis, no significant relationship was evaluated between water sorption and solubility values for each material ( *p >* 0.05).

It is important to consider that this was an *in vitro* study, so the first limitation was that the results presented in this article may not reflect a real clinical scenario. Second, the real temperatures in the patient's mouth could not be simulated. Generally, 1-week (7 days) storage time corresponds to a short-term evaluation. Therefore, longer storage times should be evaluated. Besides, the lack of the evaluation of degree of conversion and monomer elution amount, surface hardness, surface texture, wear rates, and filler–resin interface were other limitations of this study. Manual manipulation of one of the materials tested was another limitation that could alter the values. In further studies, more realistic results can be obtained with *in vivo* and clinical studies that will shed light on the clinical applications.

## Conclusion

Based on the results of the present study, it can be concluded that the most successful material in terms of water sorption was the bulk-fill nano-filled composite resin material. The bulk-fill glass hybrid restorative system was the most successful material in terms of water solubility. Alkasite, a self-adhesive restorative material, can be recommended to be used as a base material due to its high solubility feature. Monomer, filler type, and amount had an impact on the water sorption and solubility properties of the tested materials.
